# Using multi-layer perceptron to identify origins of replication in eukaryotes via informative features

**DOI:** 10.1186/s12859-021-04431-x

**Published:** 2021-10-23

**Authors:** Yongxian Fan, Wanru Wang

**Affiliations:** School of Computer Science and Information Security, Guilin University of Electronic Technology, Guilin, 541004 China

**Keywords:** Eukaryotes, DNA replication, Origin, TF-IDF, Multi-layer perceptron, STREME

## Abstract

**Background:**

The origin is the starting site of DNA replication, an extremely vital part of the informational inheritance between parents and children. More importantly, accurately identifying the origin of replication has great application value in the diagnosis and treatment of diseases related to genetic information errors, while the traditional biological experimental methods are time-consuming and laborious.

**Results:**

We carried out research on the origin of replication in a variety of eukaryotes and proposed a unique prediction method for each species. Throughout the experiment, we collected data from 7 species, including *Homo sapiens*, *Mus musculus*, *Drosophila melanogaster*, *Arabidopsis thaliana*, *Kluyveromyces lactis*, *Pichia pastoris* and *Schizosaccharomyces pombe*. In addition to the commonly used sequence feature extraction methods PseKNC-II and Base-content, we designed a feature extraction method based on TF-IDF. Then the two-step method was utilized for feature selection. After comparing a variety of traditional machine learning classification models, the multi-layer perceptron was employed as the classification algorithm. Ultimately, the data and codes involved in the experiment are available at https://github.com/Sarahyouzi/EukOriginPredict.

**Conclusions:**

The prediction accuracy of the training set of the above-mentioned seven species after 100 times fivefold cross validation reach 92.60%, 90.80%, 91.22%, 96.15%, 96.72%, 99.86%, 96.72%, respectively. It denotes that compared with other methods, the methods we designed could accomplish superior performance. In addition, our experiments reveals that the models of multiple species could predict each other with high accuracy, and the results of STREME shows that they have a certain common motif.

**Supplementary Information:**

The online version contains supplementary material available at 10.1186/s12859-021-04431-x.

## Background

**Fig. 1:**
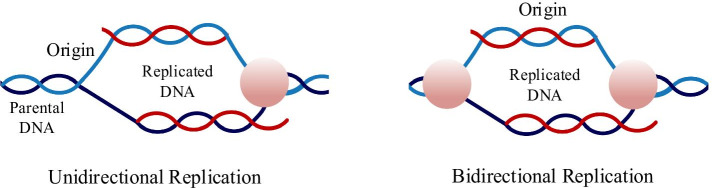
DNA replication in eukaryotes. This diagram shows the process of DNA replication in eukaryotes

DNA replication usually occurs during cell division, then two DNA molecules are distributed to daughter cells, and the genetic material is passed on to the offspring through cell proliferation. The point at which DNA commence to replicate is called the origin of replication [1]. As shown in Fig. 1, eukaryotes usually have not only one origin, and they will begin to replicate from multiple points during replication [2], which are mainly divided into unidirectional replication and bidirectional replication. Abnormal replication may result in heritable variation in the organism. The accurate replication of DNA not only maintains the continuity of genetic information, but also ensures the relative stability of the species.

However, most of related studies only focus on the organism of *Saccharomyces cerevisiae*. In 2004, Corzzareli's group [3] predicted the starting site in Saccharomyces cerevisiae by using the property of replication initiation to be rich in AT bases. In 2012, Chen et al. [4] studied the replication initiation site of Saccharomyces cerevisiae by calculating the bending degree and cleavage intensity of the DNA sequence, which is highly effective for identifying positive samples. In 2016, Zhang et al. [5] first attempted to study the origin of human DNA replication and constructed a predictor based on random forest. In 2016, Wang et al. [6] studied *H. sapiens*, *M. musculus*, *E. coli* and came up with a method “MaloPred”. The AUC values predicted by this method for these three organisms are 0.755, 0.827 and 0.871, respectively. In 2018, Liu et al. [7] studied four kinds of yeasts. In 2019, Dao et al. [8] collected a variety of eukaryotes. Based on characteristics such as Kmer and SVM classifier, they conducted a complete study of each organism and made some progress. In 2020, Wei et al. [9] presented a novel machine learning-based approach called Stack-ORI encompassing 10 cell-specific prediction models. And the prediction of origins of human and other four organisms is excellent. In consequence, it is necessary to further promote the experiment to improve the classification accuracy.

In this study, we collected datasets of 7 eukaryotes, including *Homo sapiens (H. sapiens)*, *Mus musculus (M. musculus)*, *Drosophila melanogaster (D. melanogaster)*, *Arabidopsis thaliana (A. thaliana)*, *Pichia pastoris (P. pastoris)*, *Schizosaccharomyces pombe (S. pombe)*, *Kluyveromyces lactis (K. lactis)*, and conducted independent research on each species. We employed three types of feature extraction methods (TF-IDF, PseKNC-II, Base-content), and performed the two-step feature selection method based on SVM. When selecting classification models, we compared SVM, Naïve bayes, Decision Tree, KNN, MLP, XGBoost to find the best model. In the terminate, we designed the unique classification algorithm for each organism. After the classification experiment, we conducted cross-species tests and sequence analysis using STREME [10], the results showed that there were similar motifs among various species.

## Results and discussion

### Feature analysis

**Fig. 2:**
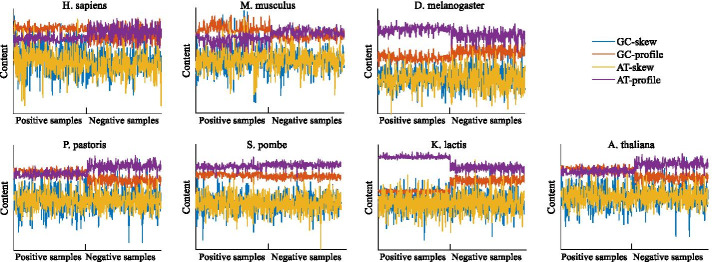
Display of Base-content. The first half of each graph corresponds to the positive sample, and the second half corresponds to the negative sample

As mentioned above, we utilized three feature extraction methods. In this chapter, we analyzed the four features of Base-content. Firstly, we randomly selected the same number of positive and negative samples from seven species, and then used the graph to describe the four characteristic values corresponding to different samples. As shown in Fig. 2, the features corresponding to the positive and negative samples of *H. sapiens*, *S. pombe* are not significantly differentiated, while the other five species have significant differences in the GC-skew and AT-profile, which indicates that the extracted features are very effective.

### Feature ranking analysis

**Fig. 3:**
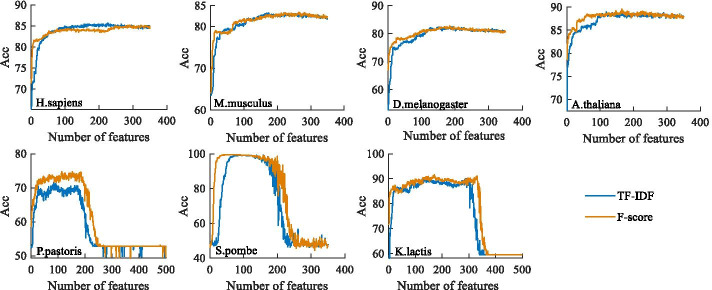
Comparison of F-score and TF-IDF. This figure shows the effect of feature selection according to the two ranking methods of F-score and TF-IDF

As mentioned above, the method we applied originally in feature ranking was F-score. However, when extracting feature TF-IDF, we found that the score of TF-IDF could also be used as the ranking standard of corresponding features. In order to compare the two methods, we respectively used the two scores as the ranking standard to carry out the IFS experiment. As shown in Fig. 3, it is wise to sort features based on TF-IDF scores and F-score, they can accurately represent the importance of features. When the number of features is small, the feature selection effect based on F-score is better, and the feature selection effect based on TF-IDF is better when the feature number is increased. For species such as *H. sapiens*, *M. musculus* and *D. melanogaster*, utilizing TF-IDF can achieve the best feature selection effect, while *A. thaliana*, *P. pastoris*, *S. pombe* and *K. lactis* are more suitable for F-score. More important, the experiment in this section could prove that feature selection significantly improves the classification effect.

### Performance evaluation on different feature extraction methods

In this experiment, we extracted three features of the sequence: TF-IDF, PseKNC-II, Base-content. By evaluating a variety of feature sets based on the SVM, we obtained the most effective feature set corresponding to each species.

In the first place, the six pseudo-nucleotide features were combined together to compare the classification effect with the single optimal nucleotide features and selected the optimal feature set as the pseudo-nucleotide feature.

**Fig. 4:**
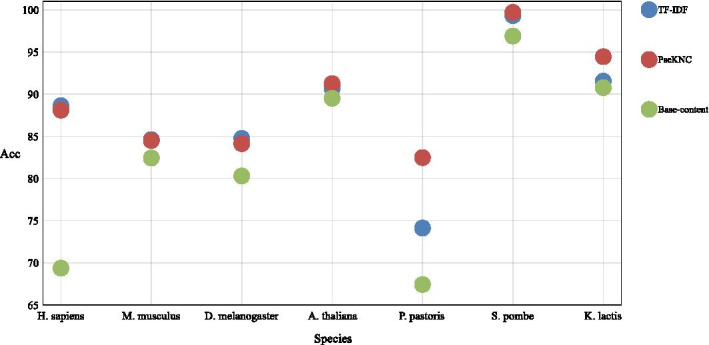
Comparison of different feature extraction methods. This figure shows the corresponding prediction effects of three feature extraction methods such as TF-IDF

After that, we compared the three feature extracted methods, as shown in Fig. 4, the features extracted by TF-IDF are the most effective for *H. sapiens*, *M. musculus*, and *D. melanogaster*; while *A. thaliana*, *P. pastoris*, *S. pombe* and *K. lactis* are more suitable for extracting pseudo-nucleotide features to represent sequences. The classification results of the specific 6 single nucleotides and combined nucleotides are shown in the Additional file 1.

### Performance evaluation on different model

**Fig. 5:**
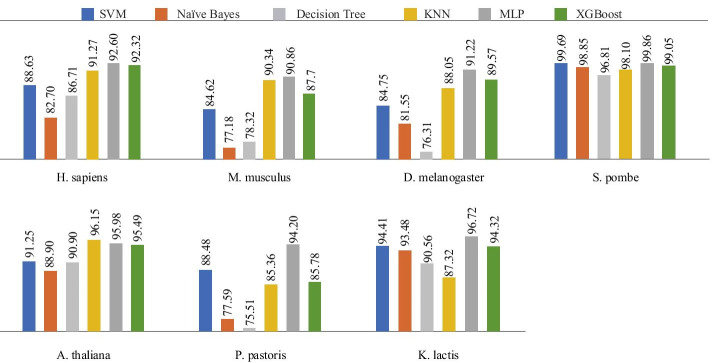
Comparison of different models. This chart shows the predictive effects of models such as MLP

In order to improve the classification accuracy as much as possible, we employed the following 6 classification models. As shown in Fig. 5, MLP is obviously superior to other models for classification of 6 species such as *H. sapiens*, and only *A. thaliana* has achieved better results on which KNN is applyed for classification.

### Comparison with published methods

In order to verify the advantages of our methods, the detailed comparison was made with the prediction methods proposed by Dao et al. [8] and Wei et al. [9] based on the same training dataset and independent test dataset. As shown in Table 1, after 100 times of fivefold cross-validation, the prediction methods we designed are much better for all species.

**Table 1:** Comparison of prediction methods based on training dataset

Species	Methods	*Acc* (%)	*Sn* (%)	*Sp* (%)	*MCC*	AUC
*H. sapiens*	Ours	92.60	91.16	94.16	0.8677	0.9983
	Wei et al. [9]	88.30	89.60	87.00	0.7660	0.9560
*M. musculus*	Ours	90.80	89.38	92.21	0.8280	0.9821
	Wei et al.	89.10	87.50	90.70	0.7662	0.9558
*D. melanogaster*	Ours	91.22	91.53	90.89	0.8219	0.9876
	Wei et al.	88.60	85.90	91.20	0.7720	0.9470
*A. thaliana*	Ours	96.15	97.07	95.17	0.9155	0.9963
	Wei et al.	94.09	93.79	93.50	0.8729	0.9817
*P. pastoris*	Ours	94.20	92.36	95.76	0.8953	0.9678
	Dao et al. [8]	88.38	87.69	89.00	0.7669	0.9500
*S. pombe*	Ours	99.86	100	100	1	0.9985
	Dao et al.	99.85	100	99.71	0.9971	0.9945
*K. lactis*	Ours	96.72	97.36	96.19	0.9251	0.9965
	Dao et al.	93.75	94.12	93.50	0.8715	0.9781

Since we only divided the datasets of *H. sapiens*, *M. musculus*, *A. thaliana* and *D. melanogaster* into training sets and independent test sets, the comparative experiments based on the independent test were only carried out for these four species. The specific results are shown in Table 2.

**Table 2:** The prediction results on test dataset

Species	Method	*Acc* (%)	*Sn* (%)	*Sp* (%)	*MCC*	AUC
*H. sapiens*	Ours	91.22	0.9153	0.9089	0.8219	0.9876
	Wei et al. [9]	87.10	0.8990	0.8420	0.7420	0.9450
*M. musculus*	Ours	89.10	0.8131	0.8430	0.6670	0.8100
	Wei et al. [9]	88	0.9160	0.8440	0.7620	0.9490
*A. thaliana*	Ours	94.20	0.9236	0.9576	0.8953	0.9678
	Wei et al.	88.80	0.9010	0.8750	0.7770	0.9480
*D. melanogaster*	Ours	90.80	0.8938	0.9221	0.8280	0.9821
	Wei et al.	87.50	0.8910	0.8590	0.7500	0.9440

### Cross-species validation and sequence analysis

**Fig. 6:**
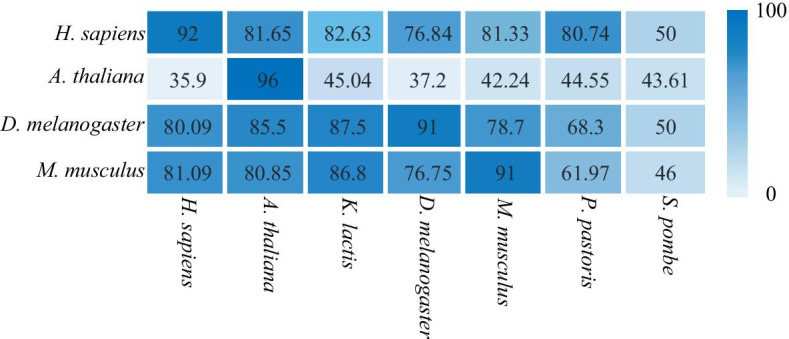
Cross-species validation. The graph shows the effect of cross-species prediction between different species

**Fig. 7:**
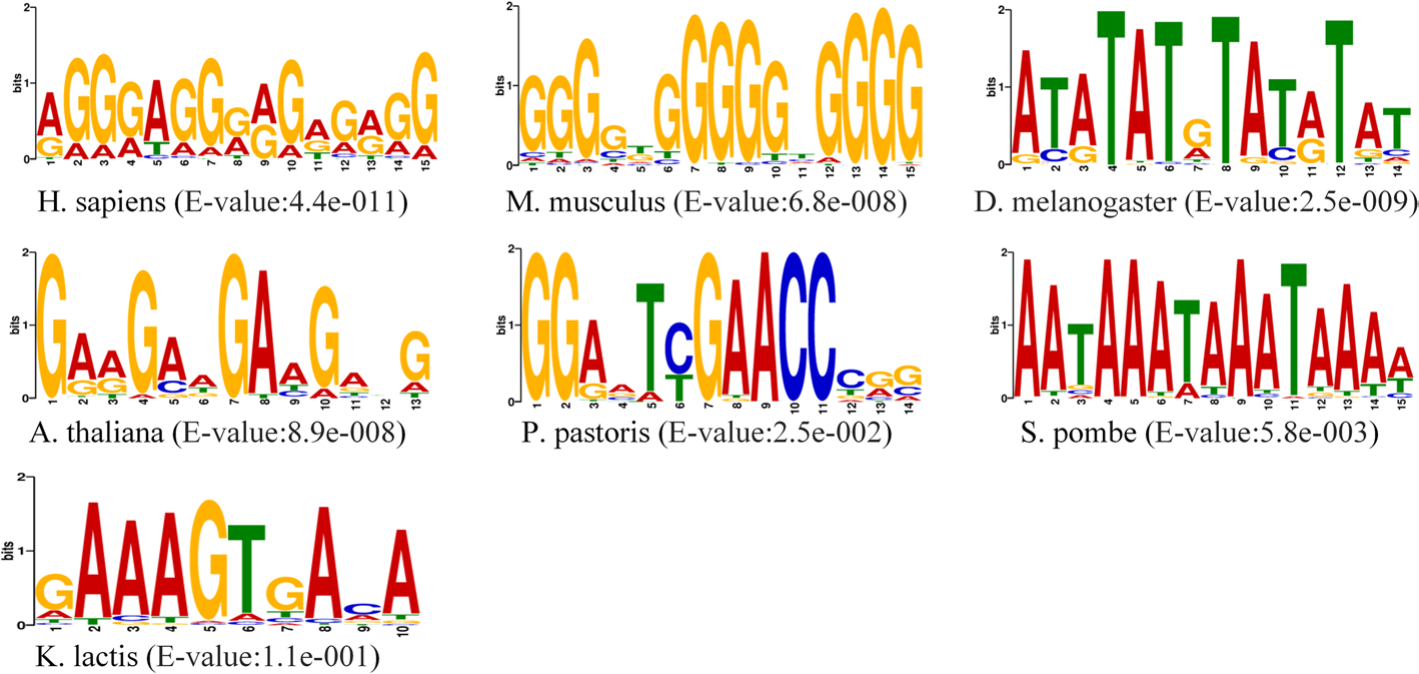
Discovered Motifs by STREME. The figure shows the motif of various species found by STREME

In this paper, we conducted independent studies on the origin of replication in seven eukaryotes and trained the corresponding models. In order to verify the predictive ability of various species models, we utilized cross-species studies. As shown in the Fig. 6, the models of *H. sapiens*, *M. musculus*, *D. melanogaster* and *A. thaliana* were employed for the classification of other species. The results shows that models of *H. sapiens*, *M. musculus, A. thaliana* and *D. melanogaster* work well in classifying other species. Then we made use of the STREME [10] to analyze the sequences, which was more suitable for processing dataset containing more than 50 sequences than MEME [11, 12]. As shown in the Fig. 7, the sequences of *H. sapiens, M. musculus* and *A. thaliana* have significantly the same motif fragment "GGG", while the sequences of *S. pombe*, *P. pastoris* and *K. lactis* have significantly the same motif fragment "AAA", which explains the high prediction accuracy in the cross-species test between *H. sapiens* and *M. musculus*, and the results of sequence analysis point out the direction for further research (Additional file 2).

## Conclusion

In this work, we studied the identification of origin of replication for seven eukaryotes. Three methods of PseKNC-II, Base-content and TF-IDF were utilized to extract features, and a variety of machine learning models were compared. Our study shows that *H. sapiens*, *M. musculus*, and *D. melanogaster* are more suitable for using TD-IDF to extract features, indicates that the algorithm of text classification is also suitable for sequence classification, and deserves further investigation. While *A. thaliana* and other three organisms using PseKNC to extract features could achieve the best classification results. After comparing various classification models, we discovered that MLP has a better classification effect for most species. In addition, the models of *H. sapiens*, *M. musculus*, and *D. melanogaster* can predict each other with high accuracy, and the results of STREME reveals that they have a certain common motif. In the terminate, we opened source the code and data employed in the experiment, hoping to provide related study with assistance.

## Methods

### The benchmark dataset

For studying the origin of DNA replication in various eukaryotes, seven sample datasets of eukaryotes were collected, which are *H. sapiens*, *M. musculus*, *D. melanogaster*, *A. thaliana*, *P. pastoris*, *S. pombe* and *K. lactis* [5, 7, 8]. Among them, all the sequences are 300 bp in length, the positive and negative sample sets are balanced on the whole. Studies indicates that the existing datasets of the three species of *H. sapiens*, *M. musculus* and *D. melanogaster* contain different cell types, despite the sample sequences of different cell types are quite different [8]. To make a distinction, we collected only one cell type sequence contained in these three species. As shown in the Table 3, benchmark datasets of *H. sapiens*, *M. musculus*, *A. thaliana* and *D. melanogaster* have more samples, consequently been divided into training set and test set in a ratio of 8:2, while dataset of the other three organisms were treated as the training set directly.

**Table 3:** The benchmark dataset

Species	Cell types	Positive	Negative	Sum
*H. sapiens*	K562	2332	2331	4663
*M. musculus*	ES	2380	2380	4760
*D. melanogaster*	Kc	6022	6000	12,022
*A. thaliana*	/	1515	1515	3030
*P. pastoris*	/	268	300	568
*S. pombe*	/	339	350	689
*K. lactis*	/	136	200	336

### Feature extraction

For sequence prediction, feature extraction is a necessary step, on account of almost all the machine learning models could only deal with numerical types [13], and it is also a considerably critical step. Extracting effective features could not only express the characteristics of the sequence in effect, but also improve the accuracy of classification using machine learning models. Since the key information extracted by different features is different, our experiments utilized a variety of feature extraction methods and carried out the comparison between TF-IDF, PseKNC-II and Base-content to capture the sequence to a variety of characteristics, raise the accuracy of the prediction.

### TF-DIF

TF-IDF [14–18] is a method proposed for text classification. The main idea is to find subject terms which appear in the text all the frequent, and these words only appear repeatedly in this type of article. Such as some common conjunctions "the" and "and", they have a higher frequency in a certain type of text, however, they are not representative, since these words are common in all articles. In general, searching common motifs for sequences is similar to the text classification. On account of that the classic algorithm TF-IDF in text classification was applied in our experiment, we made some modifications to it to extract the sequence features of DNA. The specific formula is shown as follows.

1 $$tf_{i} = \frac{{n_{i} }}{{\sum\nolimits_{i} {n_{i} } }}$$

where *tf*_*i*_ represents the frequency of the *i-*th k-tuple nucleotide in the positive sample. The value of *k* is from 1 to 6, and there are 5460 nucleotides in total, the value of *i* ranges from 1 to 5460.

2 $$IDF = \log \left( {\frac{{{|}D{|}}}{{{1} + {|}\{ j:{\text{t}}_{i} \in {\text{d}}_{j} \} {|}}}} \right)$$

where |D| represents the number of all samples, |{j: t_*i*_ ∈ d_*j*_}| represents the number of all samples containing the *i-*th k-tuple nucleotide, adding 1 to the denominator is to prevent the denominator from being 0.

3 $${\text{TF-IDF}} = {\text{TF}}*{\text{IDF}}$$

From this, the TF-IDF score corresponding to each k-tuple nucleotide could be obtained, and then a [5460 * 1] numerical matrix *L* was employed to represent each sequence and calculate the score of the corresponding position. The formula is as follows.

4 $$l_{i} = tf\_idf_{i} *n_{i}$$

Among them, *tf_idf*_*i*_ represents the TF-IDF score of the k-tuple nucleotide, and n_*i*_ represents the frequency of this nucleotide in the sequence.

### Base-content

Base-content extracts the base information of the sequence. Specifically, the content characteristics of single nucleotides (A, C, G, T) in each DNA sequence was utilized as features. Four base characteristics (GC-skew, GC-profile, AT-skew, AT-profile) were considered in this paper [3, 19–22].

5 $$AT{\text{-}}profile_{i} = \frac{{m_{i}^{{{\text{A}} + {\text{T}}}} }}{{m_{i}^{{{\text{A}} + {\text{T}} + {\text{G}} + {\text{C}}}} }}$$

6 $$GC{\text{-}}profile_{i} = \frac{{m_{i}^{{{\text{G}} + {\text{C}}}} }}{{m_{i}^{{{\text{A}} + {\text{T}} + {\text{G}} + {\text{C}}}} }}$$

7 $$GC{\text{-}}skew_{i} = \frac{{m_{i}^{{{\text{G}} - {\text{C}}}} }}{{m_{i}^{{{\text{G}} + {\text{C}}}} }}$$

8 $$AT{\text{-}}skew_{i} = \frac{{m_{i}^{{{\text{A}} - {\text{T}}}} }}{{m_{i}^{{{\text{A}} + {\text{T}}}} }}$$

Among them, $m_{i}^{{\text{G}}}$, $m_{i}^{{\text{C}}}$ represent the contents of G and C in the *i-*th sequence, respectively. $m_{i}^{{{\text{A}} + {\text{T}}}}$, $m_{i}^{{{\text{G}} + {\text{C}}}}$, $m_{i}^{{{\text{A}} + {\text{T}} + {\text{G}} + {\text{C}}}}$ each represent the content of “A + T”, “G + C” and “A + T + G + C”. $m_{i}^{{{\text{A}} - {\text{T}}}}$, $m_{i}^{{{\text{G}} - {\text{C}}}}$ represent the content of "A−T" and “G−C” individually.

### PseKNC-II

PseKNC-II, also known as the series correlation PseKNC [5, 23], which not only considers the frequency information of k-tuple nucleotides, but also calculates the physical and chemical properties of pseudo-nucleotides. In this work, we extracted three pseudo-nucleotides feature sets on which *k* = 1, 2, 3, 4, 5 and 6.

### Feature selection

When using numerous features, may confront the problem of data redundancy and the prediction accuracy will be influenced on account of the existence of invalid features. Therefore, the two-step [24, 25] method was applied to perform feature selection. The main idea is to score all the features based on F-score, and then use IFS to select the features to filter out effective features, which not only saves the calculation time on which forecasting, but also improves the accuracy of the forecast.

F-score [26] is a method of measuring the ability of a characteristic to distinguish between two classes. Given the training set *x*, set *n*^+^ and *n*^−^ to represent the number of positive samples and the number of negative samples, respectively. The F-score of the *i-*th feature could be deduced as

9 $${\text{F}}_{i} { = }\frac{{\left( {\overline{x}_{i}^{{( + )}} - \overline{x}_{i} } \right)^{{2}} + \left( {\overline{x}_{i}^{{{(} - {)}}} - \overline{x}_{i} } \right)^{{2}} }}{{\frac{{1}}{{n^{ + } - {1}}}\sum\nolimits_{{k{ = 1}}}^{{n^{ + } }} {\left( {\overline{x}_{k,i}^{{( + )}} - \overline{x}_{i}^{{( + )}} } \right)^{{2}} } + \frac{1}{{n^{ - } - {1}}}\sum\nolimits_{{k{ = 1}}}^{{n^{ - } }} {\left( {\overline{x}_{k,i}^{{{(} - {)}}} - \overline{x}_{i}^{{{(} - {)}}} } \right)^{{2}} } }}$$

where $\overline{x}_{i}$, $\overline{x}_{i}^{( + )}$, $\overline{x}_{i}^{( - )}$ represent the average value of the *i-*th feature in all samples, positive samples and negative samples, respectively. $\overline{x}_{k,i}^{( + )}$ is the *i-*th feature of the *k*th positive sample, and $\overline{x}_{k,i}^{( - )}$ is the *i-*th feature of the *k*th negative sample. The larger the F-score, the more effective this feature is.

The second step of feature selection is incremental feature selection (IFS) [24, 27]. First apply a feature as the training set, and then add the extracted feature to the training set one by one from high to low according to the scoring order of F-score and find the number of corresponding features with the highest classification accuracy at last.

### Model training

After feature selection based on SVM, the most effective feature set corresponding to each species was selected. In order to further improve the classification accuracy, 7 traditional machine learning classification models were utilized in our study, namely SVM, Decision tree, Naïve bayes [28], XGBoost, KNN and MLP. In order to compare different models with the principle of fairness and objectivity, the selected features were used to train models. Before applying different models, the vital parameters of each model need be adjusted to achieve superior performance which were evaluated by 100 times fivefold cross-validation, as shown in Table 4.

**Table 4:** Parameters and the value range of parameter adjustment

Model	Parameter	Value
SVM	c, g	[2^−5^, 2^15^] Δ = 2, [2^−15^, 2^−5^] Δ = 2^–1^
Multi-layer perceptron	alpha	0.001, 0.01, 0.1, 0.5, 1, 1.5
Decision tree	min_sample_split, max_depth	[2, 30] Δ = 2, [1, 10] Δ = 1
XGBoost	n_estimators, learning_rate	[10, 1000] Δ = 50, [0.1, 1] Δ = 0.1

Δ represents the step size

### Performance evaluation

In order to better display and compare the experimental results, the fivefold cross-validation [29] was employed on calculating the experimental results, hence more accurate results could be obtained. Evaluation parameters include *Acc*, *Sn*, *Sp*, *MCC* [30, 31]. In addition, the AUC value was also calculated through the ROC curve.

10 $$\left\{ {\begin{array}{*{20}l} {Sn = 1 - \frac{{N_{ - }^{ + } }}{{N^{ + } }}{\kern 1pt} } \hfill & {0 \le Sn \le 1} \hfill \\ {Sp = 1 - \frac{{N_{ + }^{ - } }}{{N^{ - } }}} \hfill & {0 \le Sp \le 1} \hfill \\ {Acc = 1 - \frac{{N_{ - }^{ + } + N_{ + }^{ - } }}{{N^{ + } + N^{ - } }}} \hfill & {0 \le Acc \le 1} \hfill \\ {MCC = \frac{{1 - \left( {\frac{{N_{ - }^{ + } }}{{N^{ + } }} + \frac{{N^{ - }_{ + } }}{{N^{ - } }}} \right)}}{{\sqrt {\left( {1 + \frac{{N_{ + }^{ - } - N_{ - }^{ + } }}{{N^{ + } }}} \right)\left( {1 + \frac{{N_{ - }^{ + } - N_{ + }^{ - } }}{{N^{ - } }}} \right)} }}{\kern 1pt} } \hfill & { - 1 \le MCC \le 1} \hfill \\ \end{array} } \right.$$

where *N*^+^ represents the number of origin sequences, *N*^−^ represents the number of non-origin sequences, $N_{ - }^{ + }$ represents the number of misjudged positive samples as negative samples, and $N_{ + }^{ - }$ represents the number of misjudged negative samples as positive samples.

## Supplementary Information

**Additional file 1.** PseKNC accuracy display when K changes.

**Additional file 2.** Comparison of different feature extraction methods in different species.

## Data Availability

The datasets supporting the conclusions of this article are included with article (and its Additional files). The source database of eukaryotes: http://lin-group.cn/server/iOri-Euk/download.html. Project name: EukOriginPredict. Project home page: https://github.com/Sarahyouzi/EukOriginPredict. Project inclusion: All datasets and the code needed to replicate the experiment.
